# Encapsulation of Carvedilol in Nanomicelles Improves Central Hemodynamics and Target Organ Damage Protection in Spontaneously Hypertensive Rats

**DOI:** 10.1002/prp2.70125

**Published:** 2025-06-02

**Authors:** Luciano Parola, Paula Denise Prince, Javier Alberto Walter Opezzo, Jennifer Riedel, Miguel Ángel Allo, Yanina Alejandra Santander Plantamura, Eliana P. Bin, Germán E. González, Andrea Carranza, Martín Donato, Diego A. Chiappetta, Marcela A. Moretton, Christian Höcht

**Affiliations:** ^1^ Departamento de Farmacología, Facultad de Farmacia y Bioquímica Universidad de Buenos Aires Buenos Aires Argentina; ^2^ Instituto de Tecnología Farmacéutica y Biofarmacia (InTecFyB), Facultad de Farmacia y Bioquímica Universidad de Buenos Aires Buenos Aires Argentina; ^3^ Instituto Alberto C. Taquini de Investigaciones en Medicina Traslacional (CONICET), Facultad de Medicina Universidad de Buenos Aires Buenos Aires Argentina; ^4^ Departamento de Tecnología Farmacéutica, Facultad de Farmacia y Bioquímica Universidad de Buenos Aires Buenos Aires Argentina; ^5^ Instituto de Fisiopatología Cardiovascular (INFICA), Departamento de Patología, Facultad de Medicina Universidad de Buenos Aires Buenos Aires Argentina; ^6^ Laboratorio de Patología Cardiovascular Experimental e Hipertensión Arterial, Instituto de Investigaciones Biomédicas (BIOMED UCA‐CONICET) Buenos Aires Argentina

**Keywords:** blood pressure variability, carvedilol, central blood pressure, hypertension, losartan, nanomicelles, target organ damage

## Abstract

The hypothesis of this work was that chronic treatment with carvedilol (CAR) administered in a nanomicelles‐based formulation (CAR‐NMs), which increases CAR oral bioavailability, is more effective than a conventional liquid CAR formulation (CAR‐LCF) and is comparable to chronic treatment with losartan (LOS) in improving hemodynamic parameters and preventing target organ damage (TOD) in spontaneously hypertensive (SH) rats. Chronic treatment with CAR‐NMs significantly improved central hemodynamic parameters (systolic and diastolic blood pressure (BP) and its variability) to a similar extent as LOS, and with superior efficacy than CAR‐LCF. Although LOS was more effective than CAR‐NMs and CAR‐LCF in reducing peripheral systolic BP, both LOS and CAR‐NMs, in contrast to CAR‐LCF, were able to significantly reduce short‐term BP variability indexes. Both CAR formulations and LOS significantly reduced aortic media wall thickness and interstitial collagen deposition, and lowered TNF‐α expression in left ventricle (LV) in SH rats. Only CAR‐NMs significantly reduced IL‐6 expression and were more effective in reducing ventricular TGF‐β expression in LV of SH rats. These findings suggest that encapsulation of CAR in NMs improved its ability to control central hemodynamics in SH rats when compared with CAR‐LCF, mainly due to a greater effect on carotid systolic BP and short‐term BP variability, resulting in a higher protection against TOD compared to CAR‐LCF.

**Abbreviations**
BP: blood pressure
BPV: blood pressure variation
BW: body weight
CAR: carvedilol
CAR‐LCF: carvedilol liquid conventional formulation
CAR‐NMs: Carvedilol Soluplus nanomicelles formulation
CV: coefficient of variation
D. Ant. WT: diastolic anterior wall thickness
D. Post. WT: diastolic posterior wall thickness
DBP: diastolic blood pressure
E/A: early (E) to late (A) ventricular filling velocities
EF: ejection fraction
HR: heart rate
ICF: interstitial collagen fraction
IVRT: isovolumetric relaxation time
LOS: losartan solution
LV: left ventricle
LVEDD: left ventricle end diastolic diameter
LVESD: left ventricle end systolic diameter
LVWT: left ventricle wall thickness
MBP: mean blood pressure
NMs: nanomicelles
S. Ant. WT: systolic anterior wall thickness
S. Post. WT: systolic posterior wall thickness
SBP: systolic blood pressure
SF: shortening fraction
SH: spontaneously hypertensive
TOD: target organ damage
WKY: Wistar Kyoto

## 1 Introduction

β‐blockers are widely used for the treatment of hypertension. In fact, the new Guidelines for the Management of Arterial Hypertension of the European Society of Hypertension (ESH), published in 2023, suggested switching β‐blockers to first‐line antihypertensive agents for the treatment of hypertension, placing them on equal footing with thiazide diuretics, renin angiotensin system blockers, and calcium channel blockers. This strategy would allow the use of β‐blockers as a twofer, a medicine used to treat two conditions at the same time, as this group of hypertensive drugs is also used for the treatment of other cardiovascular comorbid conditions in patients with hypertension [1, 2].

However, β‐blockers were significantly less effective than other first‐line antihypertensive drugs in preventing stroke and cardiovascular mortality in uncomplicated hypertensive patients, as was documented in placebo‐controlled randomized trials and meta‐analysis [3, 4, 5]. For instance, the Losartan Intervention for Endpoint reduction (LIFE) trial has demonstrated that losartan was superior to atenolol in reducing the incidence of major cardiovascular events in hypertensive patients, even though both drugs had a similar blood pressure (BP) lowering effect [3]. The reduced cardiovascular protection of β‐blockers compared to angiotensin receptor blockers has been attributed to their limited ability to attenuate central BP and/or BP variability (BPV) [6].

It must be acknowledged that most of the evidence regarding the lesser cardiovascular effects of β‐blockers in comparison with other first‐line antihypertensive agents in patients with uncomplicated hypertension comes from studies using the conventional non‐vasodilating β‐blockers, like atenolol or propranolol. Considering that β‐blockers exhibit heterogeneous pharmacokinetic and pharmacodynamic properties [7], the inferior efficacy of these β‐blockers with respect to other first‐line hypertensive agents should not be extended to new vasodilating β‐blockers, such as carvedilol (CAR) and nebivolol. The enhanced hemodynamic and metabolic properties of these vasodilating β‐blockers equip them with a superior ability to prevent major cardiovascular events in patients with essential hypertension compared to conventional β‐blockers. In addition, third‐generation β‐blockers also have pleiotropic, antioxidant, and anti‐inflammatory effects that could confer additional protection against target organ damage (TOD) [6]. In a previous study, we have shown the superiority of chronic treatment with third‐generation β‐blockers (CAR, nebivolol) compared to the second‐generation β‐blocker atenolol to attenuate BPV and to prevent TOD in spontaneously hypertensive (SH) rats. Our study was unique in terms of head‐to‐head comparison of the ability of third vs. second‐generation β‐blockers to attenuate BPV, suggesting that the lower cardiovascular protection of non‐vasodilating β‐blockers must not be translated to third‐generation β‐blockers in essential hypertension [8].

Although CAR has enhanced pharmacodynamic properties, its clinical efficacy is limited by its low oral bioavailability (20%–24%) and fast systemic elimination. Several pharmaceutical strategies have been developed for enhancing pharmacokinetic properties to improve cardiovascular protection of CAR. In this context, nanomicelles (NMs) are an attractive option to encapsulate water‐poorly soluble drugs, such as CAR. In a previous study, we found that CAR‐loaded poly (vinyl caprolactam)‐poly(vinyl acetate)‐poly(ethylene glycol) (Soluplus) NMs showed greater oral bioavailability than a conventional solution of CAR in Wistar normotensive rats [9].

In order to confirm if pharmacokinetic enhancement improves hemodynamic response and TOD prevention of CAR, the present study compared the effects of chronic treatment with a CAR Soluplus‐based NMs formulation with regard to a conventional liquid formulation and the first‐line antihypertensive agent losartan in male spontaneously hypertensive (SH) rats.

## 2 Materials and Methods

### 2.1 Animals

In this study, 3‐month‐old male SH rats (*n* = 36) and normotensive Wistar Kyoto (WKY) rats (*n* = 6) weighing 250–270 g were used. Animal experiments and care procedures were approved by the Animal Care Committee of the School of Pharmacy and Biochemistry of the University of Buenos Aires (EXP‐UBA No. 0037832/2019) and were in line with the Guide for the Care and Use of Laboratory Animals (NIH, 8th Ed., 2011). Animals were kept in a room at 22°C ± 2°C, with adequate air recycling and a 12‐h light/dark cycle. Animals were fed standard rodent food containing (w/w): 20% proteins, 3% fat, 2% fiber, 6% minerals, 69% starch, and vitamin supplements (Asociación Cooperativas Argentinas, Buenos Aires, Argentina).

### 2.2 Preparation of Oral Formulations

Losartan (Droguerías Saporiti, Buenos Aires, Argentina) solution (LOS) was obtained by directly diluting the drug in distilled water. Racemic CAR (R‐carvedilol: S‐carvedilol 1:1) (purity > 99%) was purchased from Droguerías Saporiti (Buenos Aires, Argentina). Due to its low water solubility, 0.5% (w/v) CAR was dissolved in a solution of 10% (w/v) ᴅ‐α‐tocopheryl polyethylene glycol 1000 succinate and 60% (w/v) propylene glycol, to obtain a CAR liquid conventional formulation (CAR‐LCF). CAR Soluplus NMs formulation (CAR‐NMs) was prepared as follows: CAR (50 mg) was dissolved in acetone (3 mL) and this solution was added dropwise to the Soluplus micellar aqueous dispersion (10 mL, 10% w/v) under magnetic stirring (100 RPM, 25°C) using a syringe infusion pump (PC11UB, APEMA, Argentina) until complete acetone evaporation (4 h). Then, the volume was adjusted to 10 mL with distilled water using a volumetric flask. Samples were filtered using 0.45 μm acetate cellulose filters (Microclar, Argentina).

### 2.3 Experimental Design

The experimental groups (*n* = 6) were determined as follows: SH rats received vehicle (SH rats), 15 mg/kg of CAR liquid conventional formulation (CAR‐LCF), 10 mg/kg losartan solution (LOS), or 15 mg/kg of CAR Soluplus NMs formulation (CAR‐NMs) by gavage once daily for 8 weeks. WKY rats were administered vehicle and were used as the normotensive control group. The doses were selected according to previous studies that evaluated the hemodynamic effects of chronic oral treatments using these drugs: 5–50 mg/kg of CAR and 10 mg/kg of losartan [10, 11, 12].

### 2.4 Indirect Determination of BP

During the last 2 weeks of treatment, the indirect determination of BP was performed. Briefly, systolic blood pressure (SBP) was determined by the tail‐cuff method using a sphygmomanometer coupled to a Grass 7C polygraph (Grass Instrument Co., Quincy, Massachusetts, USA). Before BP measurement, rats were trained three times a week for 2 weeks under the same conditions as the final measurement. The animals were conditioned in a thermostatic (28°C ± 1°C) and quiet room for 60 min and then placed in a heated acrylic restrainer at 37°C ± 1°C.

All the measurements were single blinded and performed by the same investigator. Six measurements were made for each rat during 8 different days, and the SBP was calculated as the average of the measurements of each day. The intraday fluctuation of SBP, related to short‐term BPV, was assessed by the SD of seven consecutive BP measurements during each day. Interday variation of SBP, related to mid‐term BPV, was determined by the SD of the mean SBP calculated for each day. The number of SBP measurements was based on the recommendations for BP measurement in experimental animals from the Subcommittee of Professional and Public Education of the American Heart Association Council on High Blood Pressure Research [13].

### 2.5 Direct Determination of BP

At the end of the treatment, the direct determination of carotid arterial pressure and heart rate (HR) was carried out in cannulated freely moving rats. Animals were first anesthetized with a mixture of ketamine/xylazine (35/5 mg/kg) and the left carotid artery was cannulated with a polyethylene cannula containing heparin diluted in Ringer solution (25 U/mL). The cannula was tunneled under the skin and exteriorized at the back portion of the neck. The measurements of BP were performed 24‐h after the cannula placement. For this, the cannula was connected to a Spectramed P23XL pressure transducer (Spectramed, Oxnard, California, USA) coupled to a Grass 79D polygraph (Grass Instrument Co., Quincy, MA, USA), which was connected to a digital converter adaptor unit (Polyview, PVA 1, Grass‐Astro Med, West Warwick, Rhode Island, USA). BP recordings were continuously assessed and stored for 2‐h periods using the Polyview 2.3 Astro‐Med software. SBP, diastolic blood pressure (DBP), mean arterial pressure (MAP), SD, and the coefficient of variation (CV) were estimated from whole 3‐min periods recordings. The HR was assessed from the BP recordings using the D/Dt mode of the digital signaling processing.

### 2.6 Echocardiography

Echocardiography was performed during the last week of treatment in rats anesthetized with a mixture of ketamine/xylazine (35/5 mg/kg) by means of ultrasonography with a 14‐MHz linear ultrasound transducer (Acuson Sequoia C512; Siemens Healthineers, Munich, Germany) [8]. Using leading‐edge methods and the guidelines of the American Society of Echocardiography [14], left ventricular (LV) internal dimensions and wall thickness (LVWT) were determined at systole and diastole. The LV end diastolic diameter (LVEDD) was assessed at the time of maximal LVEDD, and the LV end systolic diameter (LVESD) was estimated at the time of the most anterior systolic excursion of the posterior wall. The diastolic anterior and posterior wall thickness (D. Ant. WT and D. Post. WT, respectively), and the systolic anterior and posterior wall thickness (S. Ant. WT and S. Post. WT, respectively) were also measured. The ejection fraction (EF) and shortening fraction (SF) were calculated as parameters of systolic function. The E/A wave ratio (calculated from the early (E) to late (A) ventricular filling velocities) and the duration of the isovolumetric relaxation time (IVRT) were calculated as diastolic function indexes by the Doppler‐echo study.

### 2.7 Sample Collection

After measuring the hemodynamic and echocardiography parameters, animals were weighed and euthanized by decapitation. The LV and the thoracic aorta were removed and immediately weighed. A fraction of the LV and thoracic aorta was fixed in 10% (v/v) formaldehyde, and the rest of each tissue was immediately frozen at −80°C for subsequent processing for Western Blot determinations.

### 2.8 Tissue Histology

A fraction of LV and a section of the thoracic aorta (measuring from 5 to 10 mm above the diaphragm) were transversely cut and fixed in 10% (v/v) formaldehyde. Paraffin‐embedded sections of 5 μm of LV and thoracic aorta were cut into slices and stained with hematoxylin–eosin or Picrosirius Red. Stained sections from both tissues were evaluated under blind conditions. Sections of aorta stained with hematoxylin–eosin were analyzed using an optical microscope under 40× magnification, and the aortic media wall thickness was calculated [15]. Picrosirius Red stained images were captured at 400×, from which the interstitial collagen fraction (ICF) was calculated as previously described [8].

### 2.9 Western Blot Determinations

Samples of LV were homogenized in ice‐cold buffer (pH: 8.0) containing 150 mM NaCl, 50 mM Trizma‐HCl, 1% (v/v) sodium deoxycholate, 1 M ethylene glycol tetraacetic acid, 1 mM NaF, 1 mM phenylmethane sulfonyl fluoride, 1 mM sodium pervanadate, and 1X Halt Protease Inhibitor Cocktail (Thermo Scientific, Rockford, Illinois, USA). Samples were subjected to centrifugation at 10000 rpm for 10 min at 4°C. Supernatants were collected and their protein concentration was determined by the Lowry method [16]. Samples were resuspended in 6 x sample buffer (375 mM Tris–HCl buffer, pH 6.8 containing 12% (w/v) SDS, 50% (w/v) glycerol, 15% (v/v) β‐mercaptoethanol, and 0.06% (w/v) bromophenol blue). After heating in boiling water for 5 min, samples containing 50 μg protein were separated into a 12% SDS‐PAGE and transferred onto a polyvinylidene difluoride membrane. A solution of 3% (w/v) of non‐fat milk in PBS was used to block the membranes for 2 h at room temperature in continuous gentle shaking. Overnight incubation of the membranes with the corresponding primary antibody (1:1000 dilution in PBS) was followed by 2 h incubation with the corresponding HRP‐coupled secondary antibody (1:5000 dilution in PBS). Complexes were visualized by chemiluminescence detection using the Pierce ECL western blotting substrate, and the bands densitometries were measured using Image J Software (National Institute of Health, Bethesda, Maryland, USA). All antibodies were from Santa Cruz Biotechnology, inc. (Dallas, Texas, USA): antibody anti‐TGF‐β: sc‐130348; antibody anti‐TNF‐α: sc‐33639; anti‐IL‐6: sc‐32296; GAPDH: sc‐32233; antibody anti‐mouse IgG_1_: sc‐525408; antibody anti‐mouse IgG_2a_: sc‐542731.

### 2.10 CAR Pharmacokinetics After Oral Administration

In a separated group of SH rats, the relative bioavailability of CAR was investigated after single oral administration of CAR‐NMs (*n* = 6) or CAR‐LCF (*n* = 6) by gavage at a dose of 15 mg/kg. After drug administration, blood aliquots (70 μL) were obtained from the tail vein at different time points (0.5, 1.0, 1.5, 2, 3, 4 6, 8, 24, 28, and 32 h). The blood samples were centrifuged (10 000 rpm, 10 min, 4°C); the sera (20 μL) were deproteinized with acetonitrile (40 μL) and centrifuged (13 000 rpm, 2 min, 4°C). Drug concentration was determined by RP‐HPLC as previously reported [9]. Briefly, the analytical method consisted of a Spherisorb ODS column 5 μm, C18, 250 9 4.6 mm (Waters Spherisorb, Wexford, Ireland) and a mobile phase composed of distilled water: acetonitrile:triethylamine (55:45:0.2 v/v) adjusted to pH: 3.0 with phosphoric acid. CAR detection was performed using a fluorescence detector (FL‐3000, Thermo Finnigan, Villebon‐sur‐Yvette, France) with excitation and emission wavelengths settled at 238 and 350 nm, respectively. The drug retention time was 5.2 min using a flow rate of 1 mL/min, and the linearity range was 10–5000 ng/mL.

The following pharmacokinetic parameters were estimated by noncompartmental analysis of CAR plasma concentration profiles using the TOPFIT 2.0 program (Dr. Karl Thomae Gmbh, Schering AG, Germany): maximum plasma concentration (*C*_max_), time to the maximum plasma concentration (*t*_max_), area‐under‐the‐curve between the administration time and infinity (AUC_0–∞_), and half‐life of elimination (*t*_1/2_). Results were log transformed for statistical analysis.

### 2.11 Statistical Analysis

Statistical analyses were performed using GraphPad Prism v. 8.01 for Windows (GraphPad Software, San Diego, California, USA). Statistical analysis was applied after verification of normal distribution of the data and variables using the Kolmogorov–Smirnov test. Differences in variables among groups were tested using the unpaired *t*‐Student test or one‐way analysis of variance (ANOVA) followed by the Bonferroni test. A probability value of less than 0.05 indicated statistical significance.

## 3 Results

### 3.1 Hemodynamic Response

The analysis of indirect SBP by the tail‐cuff method showed an increase in SBP and BPV, expressed as SD or CV, in SH rats compared with normotensive WKY rats (Table 1). In SH rats, treatment with LOS, in contrast to CAR‐LCF and CAR‐NMs, significantly decreased SBP in SH rats. The analysis of both parameters of short‐term BPV, intraday SD or CV of indirect SBP, revealed a significant attenuation of BP fluctuations in SH rats treated with LOS or CAR‐NMs with regard to vehicle‐treated SH animals (Table 1). In contrast, chronic administration of CAR‐LCF was unable to reduce BPV in SH rats (Table 1).

**TABLE 1:** Indirect hemodynamic parameters of Wistar Kyoto (WKY rats) and spontaneously hypertensive rats treated with vehicle (SH rats), CAR syrup (CAR‐LCF), CAR Soluplus (CAR‐NMs), or losartan (LOS) for 8 weeks.

	WKY rats (*n* = 6)	SH rats (*n* = 6)	CAR‐LCF (*n* = 6)	CAR‐NMs (*n* = 6)	LOS (*n* = 6)
Tail‐cuff BP					
SBP (mmHg)	132 ± 5	186 ± 4*	177 ± 2*	179 ± 2*	148 ± 2* ^,^ **
Intraday SD of SBP (mmHg)	3.57 ± 0.4	9.09 ± 0.43*	8.23 ± 0.28*	6.67 ± 0.61* ^,^ **	5.79 ± 0.38* ^,^ ** ^,^ ***
CV (%)	2.72 ± 0.15	4.91 ± 0.28*	4.67 ± 0.16*	3.71 ± 0.28* ^,^ ** ^,^ ***	3.90 ± 0.11* ^,^ **
Carotid BP					
SBP (mmHg)	129 ± 8	197 ± 7*	189 ± 4*	175 ± 5* ^,^ **	178 ± 3* ^,^ **
DBP (mmHg)	93 ± 6	116 ± 3	115 ± 6	91 ± 6** ^,^ ***	106 ± 6
MBP (mmHg)	111 ± 7	163 ± 5*	155 ± 6*	135 ± 6** ^,^ ***	140 ± 3* ^,^ **
Intraday SD (mmHg)	4.38 ± 0.17	7.09 ± 0.65*	7.04 ± 0.41*	4.84 ± 0.27** ^,^ ***	4.60 ± 0.22** ^,^ ***
CV (%)	3.97 ± 0.30	4.70 ± 0.47	4.72 ± 0.30	3.59 ± 0.26** ^,^ ***	3.31 ± 0.16** ^,^ ***
HR (bpm)	363 ± 12	367 ± 8	315 ± 4* ^,^ **	333 ± 10* ^,^ **	347 ± 10***

*Note:* Data are shown as mean ± SEM. Differences in variables among groups were tested using one‐way analysis of variance (ANOVA) followed by Bonferroni test.

Abbreviations: BP, blood pressure; CV, coefficient of variation; DBP, diastolic blood pressure; HR, heart rate; MBP, mean blood pressure; SBP, systolic arterial pressure; SD, standard deviation.

* *p* < 0.05 versus WKY rats.

** *p* < 0.05 versus SH rats.

*** *p* < 0.05 versus CAR‐LCF.

Assessment of direct carotid BP revealed that treatment with LOS or CAR‐NMs, in contrast to CAR‐LCF, significantly reduced SBP compared with vehicle‐treated SH animals. CAR‐NMs was the only active treatment that diminished DBP in SH rats (Table 1). In addition, analysis of intraday SD or CV of carotid BP recordings, CAR‐NMs and LOS were more effective than CAR‐LCF for the attenuation of short‐term BPV (Table 1). Whilst CAR‐LCF and CAR‐NMs induced a reduction of HR when compared with vehicle‐treated SH animals, chronic administration of LOS did not induce a chronotropic response (Table 1).

Table 2 shows the findings from the echocardiographic evaluation of systolic and diastolic functions in the different experimental groups. When compared with vehicle‐treated SH rats, all active treatments were able to reduce D.POST.WT and D.ANT.WT. In contrast, only LOS and CAR‐NMs reduced S.POST.WT when compared with vehicle‐treated SH rats. Compared with WKY rats, SH rats treated with vehicle showed an increase in IVRT, which was prevented by chronic administration of CAR‐LCF, CAR‐NMs, or LOS. Vehicle‐treated SH rats showed an increased E/A ratio, which was reduced by CAR‐LCF but not CAR‐NMs or LOS. No differences in other echocardiographic parameters were observed (Table 2).

**TABLE 2:** Echocardiographic parameters of Wistar Kyoto (WKY rats) and spontaneously hypertensive rats treated with vehicle (SH rats), CAR syrup (CAR‐LCF), CAR Soluplus (CAR‐NMs), or losartan (LOS) for 8 weeks.

	WKY rats (*n* = 6)	SH rats (*n* = 6)	CAR‐LCF (*n* = 6)	CAR‐NMs (*n* = 6)	LOS (*n* = 6)
LVEDD (mm)	7.04 ± 0.20	6.53 ± 0.15	7.15 ± 0.20	7.09 ± 014	7.08 ± 0.12
LVESD (mm)	4.33 ± 0.15	4.05 ± 0.16	4.33 ± 0.08	4.39 ± 0.19	4.13 ± 0.11
D.POST.WT (mm)	1.60 ± 0.04	2.15 ± 0.08*	1.75 ± 0.03**	1.66 ± 0.02**	1.65 ± 0.02**
S.POST.WT (mm)	2.30 ± 0.04	2.72 ± 0.05*	2.70 ± 0.04*	2.16 ± 0.07** ^,^ ***	2.28 ± 0.04** ^,^ ***
D.ANT.WT (mm)	1.67 ± 0.04	2.28 ± 0.06*	1.63 ± 0.03**	1.64 ± 0.03**	1.68 ± 0.03**
S.ANT.WT (mm)	2.50 ± 0.08	2.73 ± 0.08	2.75 ± 0.03	2.54 ± 0.06	2.73 ± 0.05
SF (%)	37.41 ± 0.84	38.23 ± 2.42	39.51 ± 0.52	38.12 ± 0.98	34.30 ± 1.40
E/A	1.54 ± 0.09	1.85 ± 0.07*	1.55 ± 0.02**	1.67 ± 0.04	1.70 ± 0.07
IVRT (ms)	21.00 ± 0.41**	39.50 ± 2.12	23.25 ± 0.39**	25.00 ± 0.53**	26.00 ± 0.41* ^,^ **
EF (%)	75.43 ± 1.00	74.35 ± 3.24	77.51 ± 0.58	76.21 ± 1.17	71.5 ± 0.84

*Note:* Data are shown as mean ± SEM. Differences in variables among groups were tested using one‐way analysis of variance (ANOVA) followed by Bonferroni test.

Abbreviations: BW, body weight; LVW, left ventricle weight.

* *p* < 0.05 versus WKY rats.

** *p* < 0.05 versus SH rats.

*** *p* < 0.05 versus CAR‐LCF.

### 3.2 Heart Hypertrophy, Fibrosis, and Morphometry of Aorta

SH rats treated with vehicle showed a significant increase in LV mass index, expressed as LV weight/body weight (BW) ratio, compared to WKY rats. This LV hypertrophy was significantly prevented in SH rats by all the chronic treatments. However, none of the treatments were able to fully prevent LV hypertrophy, showing LV weight/BW ratios significantly higher with respect to WKY rats (Table 3). LOS and CAR‐NMs were more effective than CAR‐LCF for reducing LV hypertrophy in SH rats (Table 3).

**TABLE 3:** Left ventricular index parameters of Wistar Kyoto (WKY rats) and spontaneously hypertensive rats treated with vehicle (SH rats), CAR syrup (CAR‐LCF), CAR Soluplus (CAR‐NMs), or losartan (LOS) for 8 weeks.

	WKY rats (*n* = 6)	SH rats (*n* = 6)	CAR‐LCF (*n* = 6)	CAR‐NMs (*n* = 6)	LOS (*n* = 6)
BW (g)	417 ± 17	412 ± 15	410 ± 16	373 ± 18	375 ± 14
LVW (mg)	932 ± 32	1440 ± 58*	1210 ± 44* ^,^ **	1055 ± 39**	950 ± 58** ^,^ ***
LVW/BW	2.15 ± 0.12	3.51 ± 0.12*	3.10 ± 0.06* ^,^ **	2.82 ± 0.04* ^,^ ** ^,^ ***	2.70 ± 0.08* ^,^ ** ^,^ ***

*Note:* Data are shown as mean ± SEM. Differences in variables among groups were tested using one‐way analysis of variance (ANOVA) followed by Bonferroni test.

Abbreviations: BW, body weight; LVW, left ventricle weight.

* *p* < 0.05 versus WKY rats.

** *p* < 0.05 versus SH rats.

*** *p* < 0.05 versus CAR‐LCF.

**FIGURE 1:**
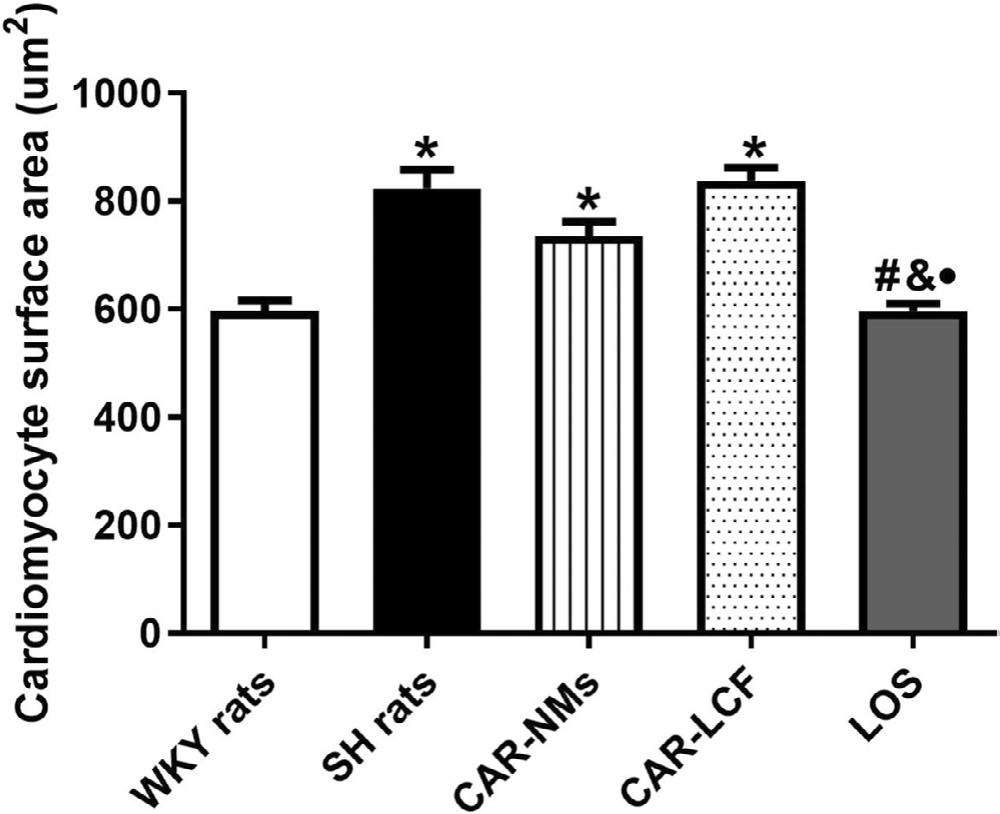
Measurement of cardiomyocyte surface area in LV slices stained with hematoxylin–eosin. Cardiomyocyte surface area in LV of Wistar Kyoto rats (WKY rats) and spontaneously hypertensive rats after 8‐week treatment with vehicle (SH rats), 15 mg/kg carvedilol‐LCF (CAR‐LCF), 10 mg/kg losartan solution (LOS), or 15 mg/kg carvedilol Soluplus‐NMs (CAR‐NMs), (*n* = 6 for all experimental groups). Data are shown as mean ± SEM. Differences in variables among groups were tested using one‐way analysis of variance (ANOVA) followed by Bonferroni test. *p < 0.05 versus WKY rats, #*p* < 0.05 versus SH rats, &*p* < 0.05 versus CAR‐LCF, •*p* < 0.05 versus CAR‐NMs. LV, left ventricle.

In the evaluation of the cardiomyocyte surface area in hematoxylin–eosin‐stained LV slices, SH rats treated with vehicle showed significantly increased cardiomyocyte surface area when compared with WKY rats. The chronic treatment with LOS also showed significantly decreased cardiomyocyte surface area with respect to SH rats treated with both CAR‐LCF and CAR‐NMs, reaching values similar to those in the normotensive control group (Figure 1). Representative images of hematoxylin–eosin‐stained LV slices from each experimental group are shown in Figure S1.

**FIGURE 2:**
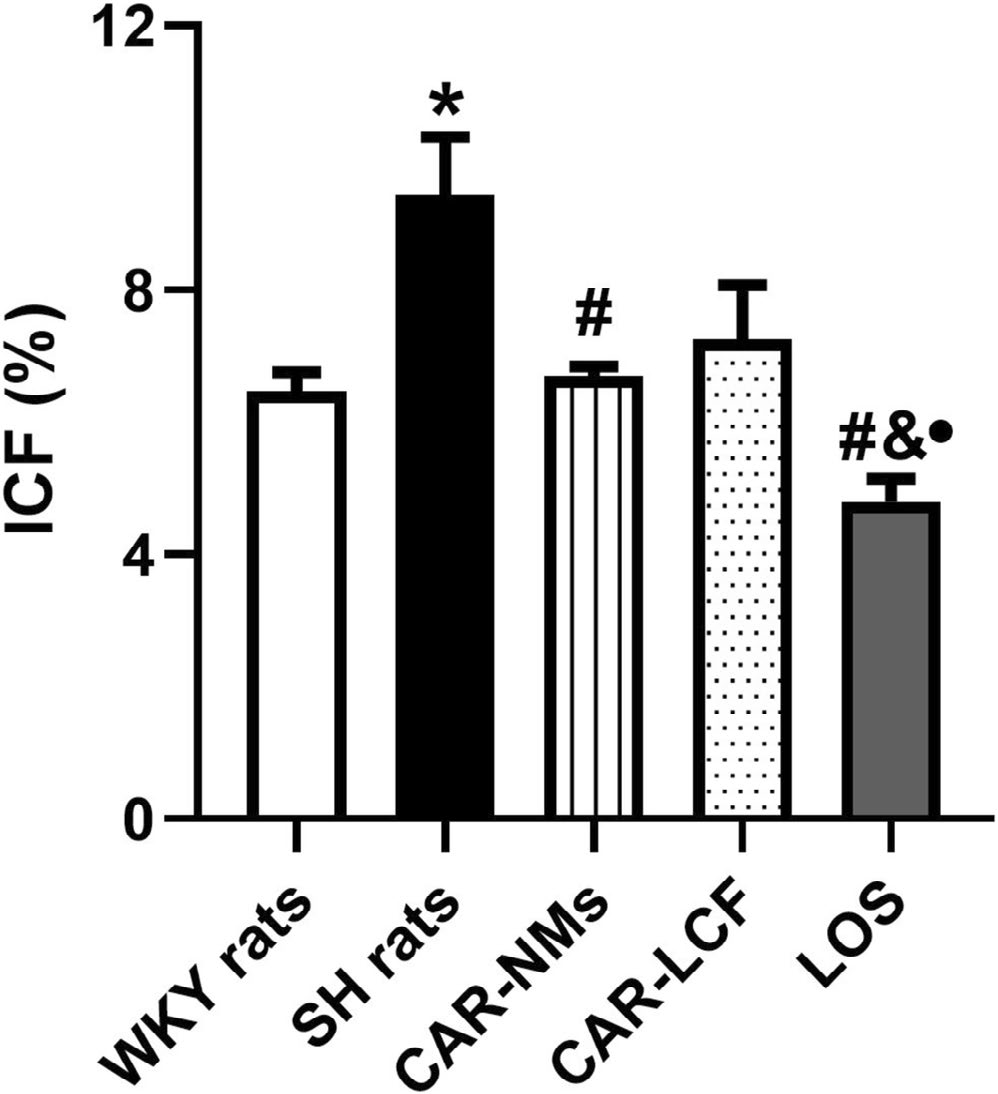
Estimation of ICF in LV slices stained with Picrosirius Red. ICF (%) in LV of Wistar Kyoto rats (WKY rats) and spontaneously hypertensive rats after 8‐week treatment with vehicle (SH rats), 15 mg/kg carvedilol‐LCF (CAR‐LCF), 10 mg/kg losartan solution (LOS), or 15 mg/kg carvedilol Soluplus‐NMs (CAR‐NMs), (*n* = 6 for all experimental groups). Data are shown as mean ± SEM. Differences in variables among groups were tested using one‐way analysis of variance (ANOVA) followed by Bonferroni test. *p < 0.05 versus WKY rats, #*p* < 0.05 versus SH rats, &*p* < 0.05 versus CAR‐LCF, •*p* < 0.05 versus CAR‐NMs. ICF, interstitial collagen fraction; LV, left ventricle.

A significant increase in interstitial myocardial collagen deposition was evidenced in SH rats treated with vehicle, determined by a significant intensification of 46% in the ICF in LV slices subjected to Picrosirius Red staining with respect to WKY rats. Chronic treatments with CAR‐NMs and LOS, but not with CAR‐LCF, were able to fully attenuate fibrosis in the LV of SH rats. Again, LOS treatment was more effective in preventing LV fibrosis than both CAR formulations, showing values of ICF significantly lower than those reached with CAR‐NMs and CAR‐LCF (Figure 2). Representative images of LV slices subjected to Picrosirius Red staining of each experimental group are shown in Figure S2.

**FIGURE 3:**
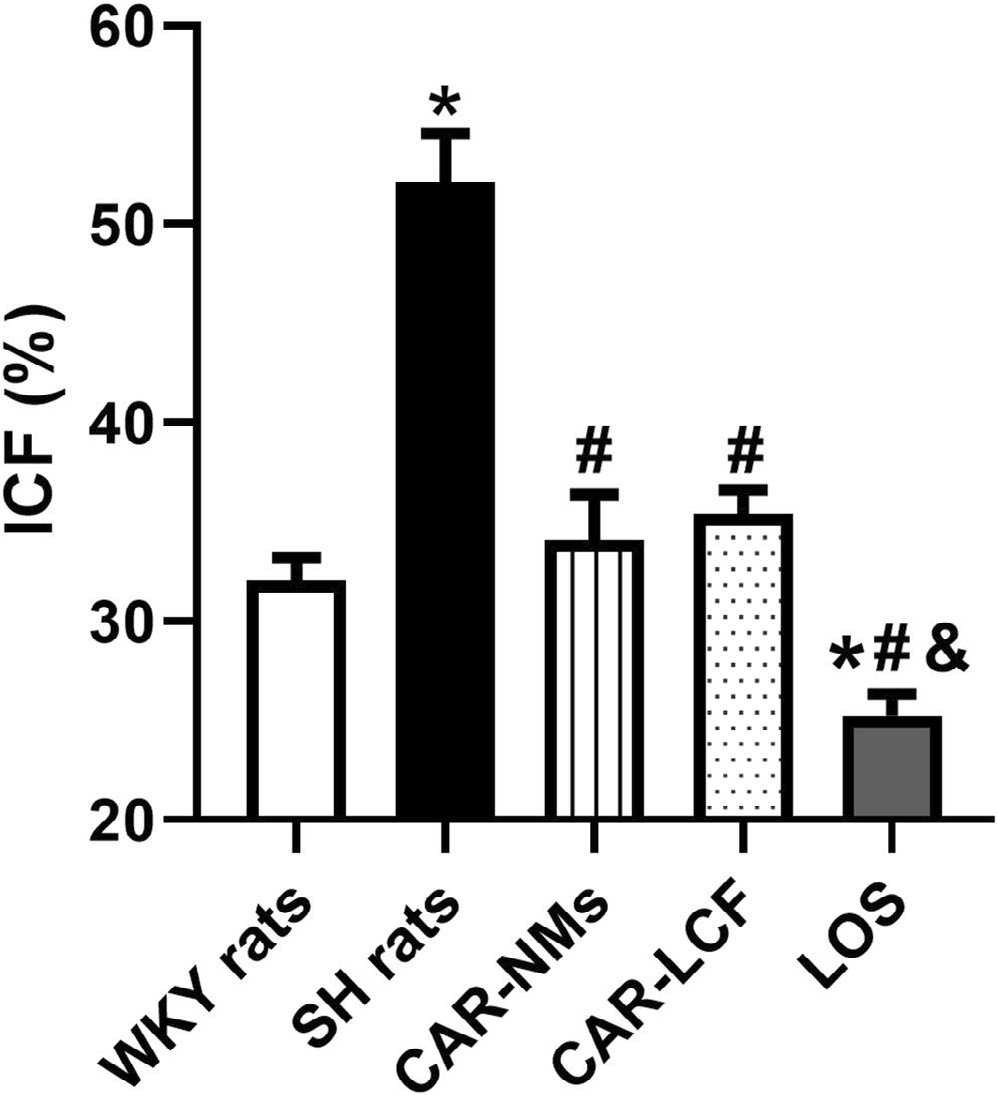
Estimation of ICF in thoracic aorta rings stained with Picrosirius Red. ICF (%) in rings of thoracic aorta from Wistar Kyoto rats (WKY rats) and spontaneously hypertensive rats after 8‐week treatment with vehicle (SH rats), 15 mg/kg carvedilol‐LCF (CAR‐LCF), 10 mg/kg losartan solution (LOS), or 15 mg/kg carvedilol Soluplus‐NMs (CAR‐NMs), (*n* = 6 for all experimental groups). Data are shown as mean ± SEM. Differences in variables among groups were tested using one‐way analysis of variance (ANOVA) followed by Bonferroni test. *p < 0.05 versus WKY rats, #*p* < 0.05 versus SH rats, &*p* < 0.05 versus CAR‐LCF. ICF, interstitial collagen fraction; LV, left ventricle.

In transversal aortic rings stained with Picrosirius Red, SH rats treated with vehicle showed a significant increase in ICF, by almost 63%, compared to WKY rats. Chronic treatments with CAR‐NMs, CAR‐LCF, and LOS completely prevented increased fibrosis in the aorta of SH rats, reaching with LOS treatment values of ICF even lower than in the normotensive control group (Figure 3). Representative images of transversal aortic rings subjected to Picrosirius Red from each experimental group are shown in Figure S3.

**FIGURE 4:**
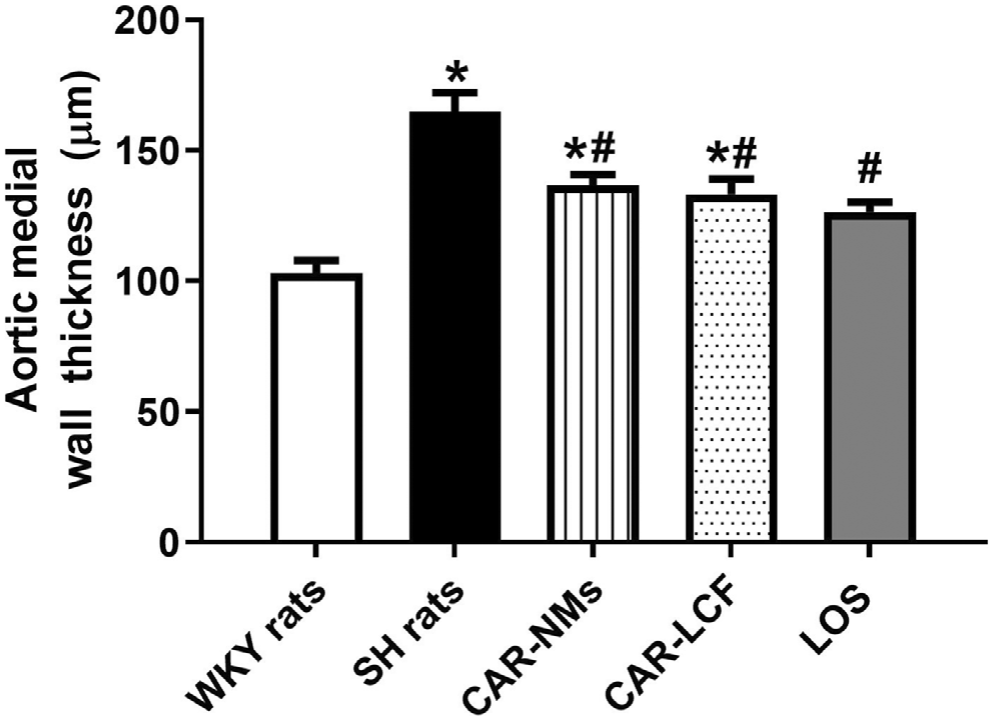
Estimation of thoracic aorta wall thickness in aorta rings stained with hematoxylin–eosin. Aortic wall thickness of Wistar Kyoto rats (WKY rats) and spontaneously hypertensive rats after 8‐week treatment with vehicle (SH rats), 15 mg/kg carvedilol‐LCF (CAR‐LCF), 10 mg/kg losartan solution (LOS), or 15 mg/kg carvedilol Soluplus‐NMs (CAR‐NMs), (*n* = 6 for all experimental groups). Data are shown as mean ± SEM. Differences in variables among groups were tested using one‐way analysis of variance (ANOVA) followed by Bonferroni test. *p < 0.05 versus WKY rats, #*p* < 0.05 versus SH rats.

The analysis of the morphology of aorta sections stained with hematoxylin–eosin showed a significant increase in the aortic medial wall thickness in SH rats treated with vehicle in comparison to normotensive WKY rats. Chronic treatment with CAR‐LCF, CAR‐NMs, and LOS significantly reduced this alteration of the aortic wall. In SH rats receiving chronic treatment with LOS, values of aortic medial wall thickness were significantly lower than those in CAR‐NMs and CAR‐LCF, and similar to those in WKY rats (Figure 4). Representative images of aortic sections stained with hematoxylin–eosin from each experimental group are shown in Figure S4.

### 3.3 Inflammation and Fibrosis Markers in LV

**FIGURE 5:**
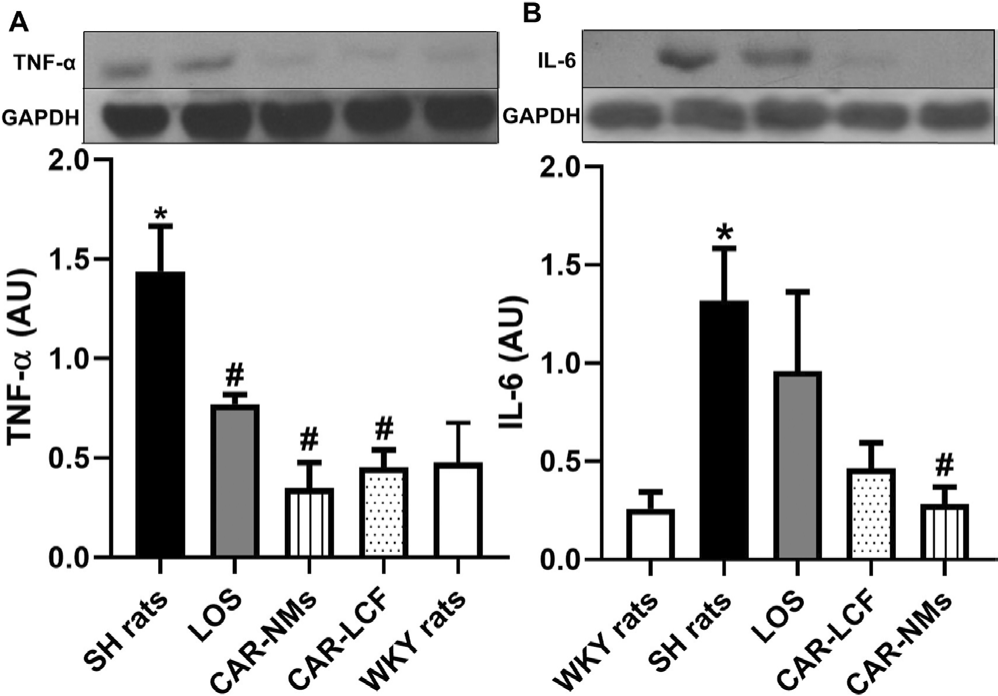
Proinflammatory cytokines expression levels in LV measured by Western Blot. Levels of TNF‐a (A) and IL‐6 (B) in the LV of Wistar Kyoto (WKY rats) and spontaneously hypertensive rats after 8‐week treatment with vehicle (SH rats), 15 mg/kg carvedilol‐LCF (CAR‐LCF), 10 mg/kg losartan solution (LOS), or 15 mg/kg carvedilol Soluplus‐NMs (CAR‐NMs), (*n* = 6 for all experimental groups). Data are shown as mean ± SEM. Differences in variables among groups were tested using one‐way analysis of variance (ANOVA) followed by Bonferroni test. *p < 0.05 versus WKY rats, #*p* < 0.05 versus SH rats. AU, arbitrary units; LV, left ventricle.

The levels of biochemical markers of TOD were assessed in LV. Both inflammatory biomarkers were significantly increased in SH rats with respect to WKY rats. TNF‐α expression levels were significantly decreased in LV of SH rats receiving chronic treatment with LOS, CAR‐LCF, or CAR‐NMs, reaching values similar to those in WKY rats (Figure 5A). Increased IL‐6 expression levels in SH rats with respect to the WKY rats could only be significantly lowered by the chronic treatment with CAR‐NMs, reaching values similar to those in WKY rats. In SH rats chronically treated with CAR‐LCF, a decreased expression of IL‐6 was evidenced, but it did not reach statistical significance (Figure 5B).

**FIGURE 6:**
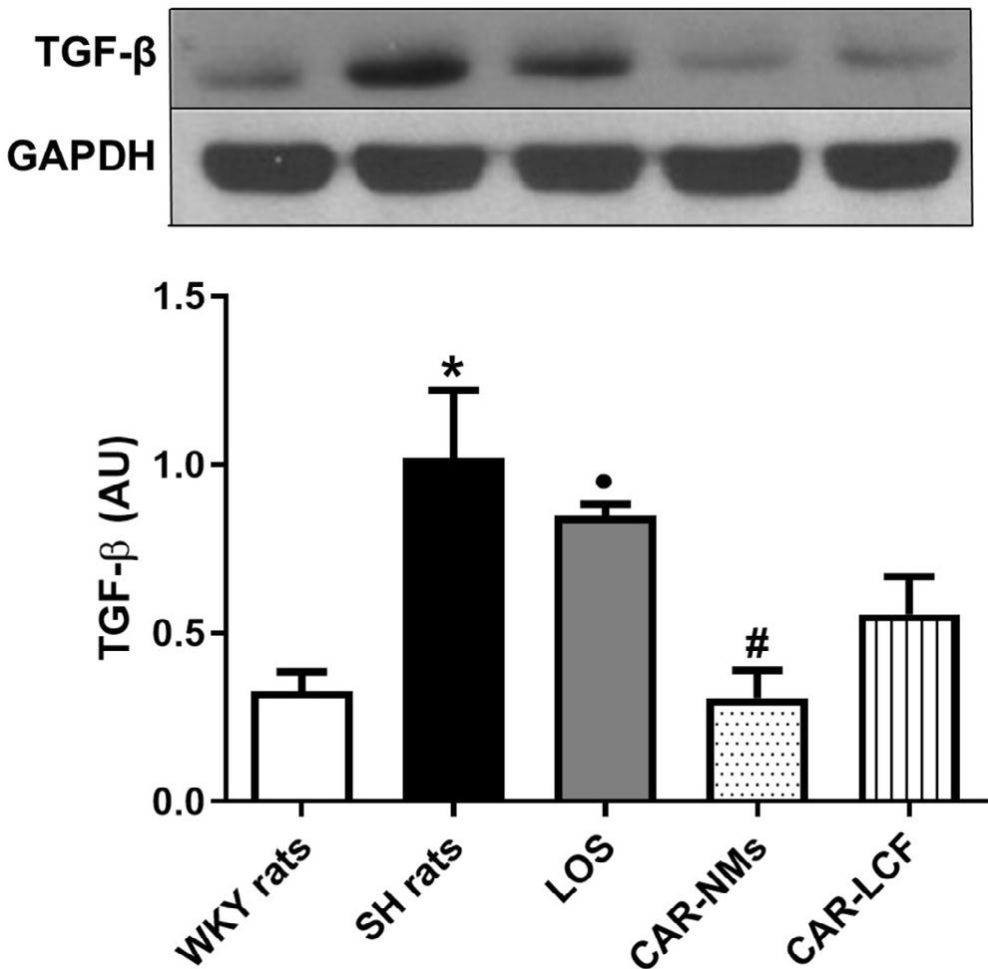
TGF‐β expression levels in the LV measured by Western Blot. Levels of TGF‐β in the LV of Wistar Kyoto (WKY rats) and spontaneously hypertensive rats after 8‐week treatment with vehicle (SH rats), 15 mg/kg carvedilol‐LCF (CAR‐LCF), 10 mg/kg losartan solution (LOS), or 15 mg/kg carvedilol Soluplus‐NMs (CAR‐NMs), (*n* = 6 for all experimental groups). Data are shown as mean ± SEM. Differences in variables among groups were tested using one‐way analysis of variance (ANOVA) followed by Bonferroni test. *p < 0.05 versus WKY rats, #*p* < 0.05 versus SH rats, •*p* < 0.05 versus CAR‐NMs. AU, arbitrary units; LV, left ventricle.

The expression of the profibrotic marker TGF‐β was significantly increased in LV of SH rats compared to the normotensive control group. Only chronic treatment with CAR‐NMs significantly decreased the expression of TGF‐β in LV from SH rats, reaching values similar to those in WKY rats. Moreover, values of TGF‐β in the LV from SH rats chronically treated with CAR‐NMs or CAR‐LCF were significantly lower than those from SH rats treated with LOS (Figure 6).

### 3.4 Pharmacokinetics of CAR After Oral Administration of CAR‐NMs or CAR‐LCF

**FIGURE 7:**
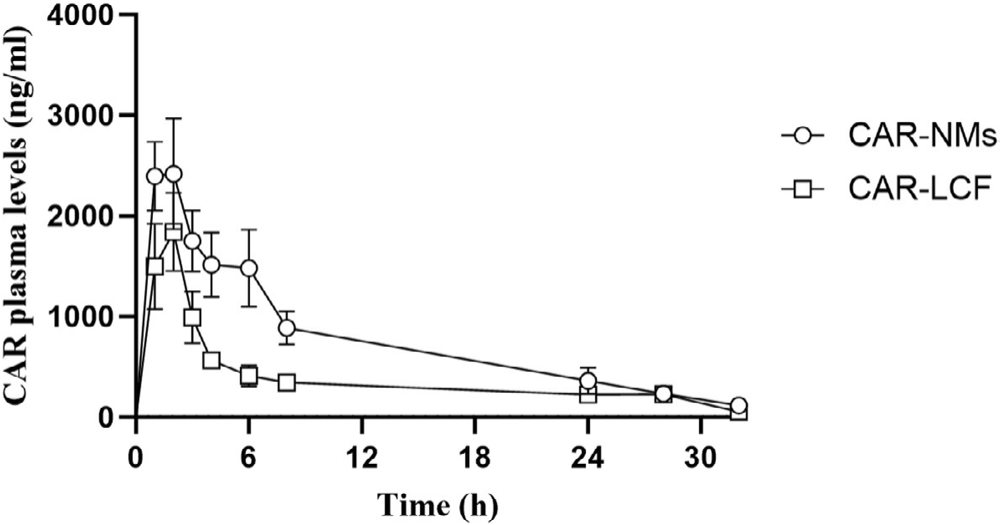
CAR plasma levels determined by RP‐HPLC. Plasma levels of carvedilol were measured after a single oral administration of 15 mg/kg CAR‐LCF or CAR‐NMs. Data are shown as mean ± SEM.

Figure 7 shows the mean plasma levels of CAR after a single oral administration of CAR‐NMs or CAR‐LCF at a dose of 15 mg/kg. For most of the 24‐h period, CAR plasma levels were significantly greater after the intake of CAR‐NMs compared with CAR‐LCF (Figure 7). The main pharmacokinetic parameters obtained from the plasma profile of CAR are shown in Table 4. Although no differences were found in *C*_max_, *t*_max_, and *t*_1/2_ when comparing both CAR formulations, the AUC_0–∞_ was 1.84‐fold higher after the oral administration of CAR‐NMs than CAR‐LCF (Table 4).

**TABLE 4:** Pharmacokinetic parameters of CAR after single oral administration of CAR‐LCF or CAR‐NMs at a dose of 15 mg/kg.

Pharmacokinetic parameter	CAR‐LCF (*n* = 6)	CAR‐NMs (*n* = 6)
*C*_max_ (ng/mL)	2141 ± 342	2942 ± 334
*t*_max_ (h)	1.60 ± 0.22	2.67 ± 0.80
AUC_0–∞_ (ng/mL*h)	13 726 ± 1183	25 364 ± 4102*
*t*_1/2_ (h)	8.80 ± 2.01	7.53 ± 0.56

*Note:* Data are shown as mean ± SEM. Differences in variables among groups were tested using unpaired *t*‐Student test.

Abbreviations: AUC_0–∞_, area‐under‐the‐curve between the administration time and infinity; CAR, carvedilol; CAR‐LCF, carvedilol liquid conventional formulation; CAR‐NMs, carvedilol Soluplus nanomicelles; *C*_max_, maximum plasma concentration; *t*_1/2_, half‐life of elimination; *t*_max_, time to the maximum plasma concentration.

* *p* < 0.05 versus CAR‐LCF.

## 4 Discussion

The current study was designed to evaluate the relative efficacy of CAR, administered in two different formulations, with respect to the first‐line antihypertensive drug LOS for the control of hemodynamic parameters and the prevention of TOD in SH rats, a classical model of high BP, characterized by increased BP as well as BPV, cardiac hypertrophy, vascular remodeling, and renal damage [17, 18, 19]. In the present work, we showed that chronic treatment with CAR‐NMs was able to improve central hemodynamics and to reduce BPV in SH rats to a similar extent as LOS, providing a comparable TOD protection throughout echocardiographic, macroscopic, histological, and biochemical parameters. Moreover, CAR‐NMs were shown to be superior to CAR‐LCF in terms of central BP and BPV reduction, as well as attenuation of LV hypertrophy in SH rats.

β‐blockers have been associated with a reduced ability to prevent or regress cardiovascular damage, including LV hypertrophy, carotid intima–media thickness, and aortic stiffness, possibly due to their lower ability to reduce central BP and BPV. Nevertheless, it is a well‐known fact that β‐blockers differ in their pharmacokinetic and pharmacodynamic properties, and vasodilatory third‐generation β‐blockers, that is, CAR, show a greater ability to improve hemodynamic alterations associated with hypertension. In a previous study, we have shown that chronic treatments with CAR or nebivolol induce a greater attenuation of BPV than atenolol in SH rats [8]. Moreover, the hemodynamic response to chronic administration of CAR or nebivolol was comparable to amlodipine in terms of improvement of carotid BP and attenuation of short‐term BPV in N(v)‐nitro‐L arginine methyl ester (L‐NAME)‐induced hypertensive rats [20]. In order to expand the comparison of third‐generation β‐blockers with other first‐line antihypertensive drugs, in the present study, we assessed the BP lowering efficacy of CAR‐NMs, CAR‐LCF, and LOS by means of direct and indirect measurements. Although LOS was more effective than CAR‐NMs and CAR‐LCF in reducing peripheral SBP, both LOS and CAR‐NMs, in contrast to CAR‐LCF, were able to reduce short‐term BPV indexes in SH rats. These findings suggest that encapsulation of CAR in NMs increases the ability of the β‐blocker to attenuate BPV obtained from tail‐cuff measurements. In fact, in a previous study, an increased half‐life of CAR after oral administration of CAR‐NMs has been demonstrated when compared with CAR‐LCF, supporting their increased ability to control BPV associated with the hypertensive stage in SH rats [9].

In addition, the analysis of carotid BP measurements confirmed that chronic treatment with CAR‐NMs improved central hemodynamics in SH rats in a similar manner to LOS, reducing both SBP, DBP, and its variability. Moreover, at the studied dose, CAR‐NMs were superior to CAR‐LCF in reducing DBP, MAP, and short‐term BPV in SH rats, confirming that drug encapsulation in polymeric NMs enhances long‐term cardiovascular response to CAR. In fact, in the present study, chronic treatment with CAR‐LCF 15 mg/kg once‐daily induced a non‐significant reduction of peripheral and carotid BP when compared with vehicle‐treated SH rats, contrasting with the significant BP response reported with CAR‐LCF 30 mg/kg once‐daily in a previous report [8]. In order to establish whether the enhanced hemodynamic response to CAR after CAR‐NMs could be explained by increased drug exposure, we assessed the relative oral bioavailability of CAR after a single administration of CAR‐NMs or CAR‐LCF in a separate experiment. The pharmacokinetic analysis demonstrated that CAR‐NMs increase oral bioavailability of CAR by 84% when compared with CAR‐LCF, suggesting that the enhanced cardiovascular response to CAR‐NMs could be explained, at least in part, by an increased exposure to CAR during a chronic administration.

In addition, although both CAR formulations were able to reduce the HR in SH rats, only CAR‐LCF induced a greater HR reduction when compared with LOS treated animals. The lower change in HR reported with CAR‐NMs can be attributed to a greater reduction in peripheral vascular resistance secondary to a greater drug exposure. Previous studies have shown that CAR is able to induce a lower decrease in HR than other non‐vasodilatory β‐blockers due to an increase in sympathetic tone as a physiological reaction to the blood pressure lowering effect of α‐blockade [21]. Although HR reduction was numerically lower in SH rats treated with CAR‐NMs than with CAR‐LCF, it seems that this finding is not relevant for the blood pressure lowering response, considering that any decrease in HR may be compensated by an increase in stroke volume, which will diminish the effect on SBP [22].

The comparable ability of CAR‐NMs and LOS to control central hemodynamics in SH rats in our report is in line with the findings from a previous clinical study that assessed the relative efficacy of CAR and LOS in 201 hypertensive patients. The prospective, randomized, and multicenter clinical study demonstrated that oral treatment with CAR or LOS for 24 weeks showed comparable effects on central hemodynamic indices, inflammatory parameters, and peripheral BP [23].

Echocardiographic findings showed that all active treatments improved some parameters of cardiac structure or function with subtle differences among experimental groups. Of relevance, CAR‐NMs and LOS normalized S.POST.WT to values reported in WKY normotensive rats and showed greater efficacy than CAR‐LCF. The greater efficacy of CAR‐NMs and LOS in reducing S.POST.WT in SH rats could be explained by their enhanced hemodynamic response, considering that S.POST.WT is associated with higher SBP [24]. In fact, S.POST.WT represents an early echocardiographic marker, and it is present in stage I hypertension [25]. Of note, CAR‐NMs showed similar efficacy in reducing S.POST.WT to LOS, a highly effective first‐line antihypertensive agent. Findings from a clinical trial have demonstrated that losartan was more effective than amlodipine in LV PWT in hypertensive patients [26].

In the present work, we also aimed to assess the effects of chronic treatment with both CAR formulations or LOS on TOD at the LV and the thoracic aorta of SH rats. Hypertension is strongly associated with increased circulating levels of proinflammatory cytokines such as IL‐1β, IL‐6, and TNF‐α [27]. Urinary excretion of TNF‐α has been proposed as a marker of TOD in patients with essential hypertension, as it correlated with urinary albumin excretion and Cornell product, two markers of renal and heart failure, respectively [28]. In parallel, IL‐6 and C reactive protein plasma levels were associated with BPV in hypertensive patients, suggesting systemic inflammation as a pathophysiological mechanism contributing to TOD in hypertension [29]. The pathophysiological mechanisms of LV hypertrophy induced by a hypertensive state include impaired diastolic and systolic ventricular function, increased interstitial collagen deposition, and overexpression of the profibrotic cytokine TGF‐β [27]. In SH rats, levels of IL‐1β, IL‐6, and TNF‐α in LV and thoracic aorta were higher compared to the normotensive WKY rats and correlated with LV hypertrophy and the profibrotic cytokine TGF‐β expression in both tissues [8, 30]. We have previously demonstrated the protective effect of chronic oral treatment with the vasodilating β‐blockers CAR and nebivolol on TOD, and their superiority compared to the non‐vasodilating β‐blocker atenolol. Increased medial wall thickness of the thoracic aorta, collagen deposition, and expression of TNF‐α, IL‐6, and the profibrotic cytokine TGF‐β in LV and thoracic aorta were significantly lower in SH rats receiving chronic oral treatment with CAR or nebivolol. Both vasodilating β‐blockers showed superiority when preventing TOD compared to atenolol, reducing myocardial damage to a similar extent as amlodipine [8]. In line with this work, rats with acute myocardial infarction had a reduction of hypertrophy and myocardial fibrosis, with decreased levels of TGF‐β expression when receiving a 4‐week therapy with CAR starting 24 h after infarction [31].

In the present work, chronic treatment with both CAR formulations and LOS had a protective effect against TOD in the thoracic aorta, reducing aortic media wall thickness and interstitial collagen deposition. Therefore, it seems that differences in the enhanced central hemodynamic response to CAR after chronic treatment with NMs when compared to LCF do not contribute to a greater prevention of vascular remodeling in SH rats.

In LV, both formulations had the same significant lowering effect on TNF‐α expression as LOS, but only CAR‐NMs were able to significantly reduce IL‐6 expression among the three active treatments. According to these results, CAR‐NMs had a more evident anti‐inflammatory effect in LV of SH rats. Although a reduction in ventricular expression of IL‐6 by chronic LOS treatment was expected, previous findings from other authors suggest that the effect of LOS on IL‐6 expression depends on dose and treatment duration. In fact, Andrzejczak et al. have demonstrated that LOS is more effective in reducing lipopolysaccharide (LPS)‐induced serum concentrations of TNF‐α than of IL‐6 [32].

In addition, CAR‐NMs were more effective than CAR‐LFC and LOS in reducing ventricular TGF‐β expression in SH rats, allowing the achievement of cytokine levels similar to those detected in WKY normotensive animals. In other words, encapsulation of CAR in NMs enhanced the efficacy of the β‐blocker in reducing the expression of ventricular TGF‐β, contributing to a greater ability to prevent myocardial fibrosis in SH rats. In this regard, CAR‐NMs, in contrast to CAR‐LCF, were able to reduce ICF in the left ventricle of SH rats, demonstrating greater protection from cardiac remodeling. Although chronic treatment with LOS was ineffective in reducing ventricular TGF‐β expression in SH rats, it showed remarkable activity to control ICF deposition. Therefore, our results suggest that the antifibrotic effect of LOS relies more on the control of other mechanisms involved in ventricular collagen deposition, including reduction of TIMP‐1 gene expression. Previous studies have demonstrated that chronic treatment with LOS can prevent angiotensin II‐induced overexpression of cardiac TGF‐β, even though the magnitude of this effect is limited [33]. In addition, it has been reported that LOS is able to prevent the cardiac up‐regulation of tissue inhibitor of metalloproteinases 1 (TIMP‐1) gene expression, restoring the collagenolytic activity in SH rats, a mechanism that contributes to reducing ventricle fibrosis [34].

Finally, in our study, active treatments differed in their ability to reduce LV hypertrophy, considering that only CAR‐NMs and LOS were able to prevent cardiac hypertrophy in SH rats. Therefore, our results suggest that encapsulation of CAR in NMs increased the efficacy of the β‐blocker to prevent cardiac TOD when compared with CAR‐LCF, resulting in a similar protective effect to the first‐line antihypertensive agent LOS. Our findings also demonstrate that the reduction of LV hypertrophy in CAR‐treated SH rats is mainly driven by the attenuation of myocardial collagen deposition rather than the reduction of cardiomyocyte hypertrophy. Although it is well known that CAR is able to control both cardiomyocyte hypertrophy and myocardial fibrosis in SH rats [35], it seems that at the studied dose the effect of CAR on the prevention of myocardial collagen deposition predominates. The greater effect of CAR on myocardial fibrosis could also be explained by the fact that vehicle‐treated SH rats showed a greater increase in myocardial fibrosis than in cardiomyocyte hypertrophy when compared with normotensive WKY rats. It can be inferred that the greater central hemodynamic response and the enhanced ability to reduce cardiac expression of TGF‐β have contributed to the higher prevention of cardiac TOD in SH rats when compared with CAR‐LCF.

In conclusion, the main findings of our study suggest that encapsulation of CAR in NMs increased oral bioavailability and improved the ability of the vasodilating β‐blocker to control central hemodynamics in SH rats when compared with CAR‐LCF, mainly due to a greater effect on carotid SBP and short‐term BPV. As a result, CAR‐NMs were more effective than CAR‐LCF in reducing LV fibrosis and cardiac hypertrophy in SH rats, allowing a TOD prevention similar to that achieved with the first‐line antihypertensive drug LOS. These findings reinforce the fact that the lower cardioprotection in uncomplicated arterial hypertension described for conventional nonvasodilating β‐blockers must not be extrapolated to vasodilating agents, such as CAR.

## Author Contributions

C.H. and M.A.M. conceived and designed the experiments. D.A.C. and J.R. prepared the carvedilol formulations. L.P., P.D.P., and A.C. performed the experiments. J.A.W.O. measured and quantified plasma carvedilol concentrations. M.Á.A. and Y.A.S.P. measured the hemodynamic parameters. G.E.G. made the histologic analysis. E.P.B. and M.D. performed the echocardiographic analysis. L.P. and P.D.P. wrote the manuscript with input from all authors. All authors discussed the results and commented on the manuscript.

## Ethics Statement

The animal care procedures experiments were approved by the Animal Care Committee of the School of Pharmacy and Biochemistry of the University of Buenos Aires (EXP‐UBA No. 0037832/2019) and were in line with the Guide for the Care and Use of Laboratory Animals (NIH, 8th Ed., 2011).

## Conflicts of Interest

The authors declare no conflicts of interest.

## Supporting information


**Figure S1.** Representative images of LV slices stained with hematoxylin–eosin. Hematoxylin–eosin staining was used for the analysis of cardiomyocyte surface area in LV of Wistar Kyoto rats (WKY rats) and spontaneously hypertensive rats after 8‐week treatment with vehicle (SH rats), 15 mg/kg carvedilol‐LCF (CAR‐LCF), 10 mg/kg losartan solution (LOS), or 15 mg/kg carvedilol Soluplus‐NMs (CAR‐NMs). (Original magnification 400×). LV, left ventricle.

**Figure S2.** Representative images of LV slices stained with Picrosirius Red. Picrosirius Red staining was used for the estimation of ICF in LV of Wistar Kyoto rats (WKY rats) and spontaneously hypertensive rats after 8‐week treatment with vehicle (SH rats), 15 mg/kg carvedilol‐LCF (CAR‐LCF), 10 mg/kg losartan solution (LOS), or 15 mg/kg carvedilol Soluplus‐NMs (CAR‐NMs). (Original magnification 400×). ICF, interstitial collagen fraction; LV, left ventricle.

**Figure S3.** Representative images of thoracic aorta rings stained with Picrosirius Red. Picrosirius Red staining was used for the estimation of ICF in thoracic aorta from Wistar Kyoto rats (WKY rats) and spontaneously hypertensive rats after 8‐week treatment with vehicle (SH rats), 15 mg/kg carvedilol‐LCF (CAR‐LCF), 10 mg/kg losartan solution (LOS), or 15 mg/kg carvedilol Soluplus‐NMs (CAR‐NMs). (Original magnification 400×). ICF, interstitial collagen fraction.

**Figure S4.** Representative images of thoracic aorta rings stained with hematoxylin–eosin. Hematoxylin–eosin staining was used for the estimation of aortic wall thickness in Wistar Kyoto rats (WKY rats) and spontaneously hypertensive rats after 8‐week treatment with vehicle (SH rats), 15 mg/kg carvedilol‐LCF (CAR‐LCF), 10 mg/kg losartan solution (LOS), or 15 mg/kg carvedilol Soluplus‐NMs (CAR‐NMs). (Original magnification 40×).

## Data Availability

All data will be available for the reviewers if requested.
