# Supplementary figures and images for: Encapsulation of Carvedilol in Nanomicelles Improves Central Hemodynamics and Target Organ Damage Protection in Spontaneously Hypertensive Rats
Source: Pharmacol Res Perspect. 2025 Jun 2;13(3):e70125. doi: 10.1002/prp2.70125 (PMC12127884; doi:10.1002/prp2.70125)

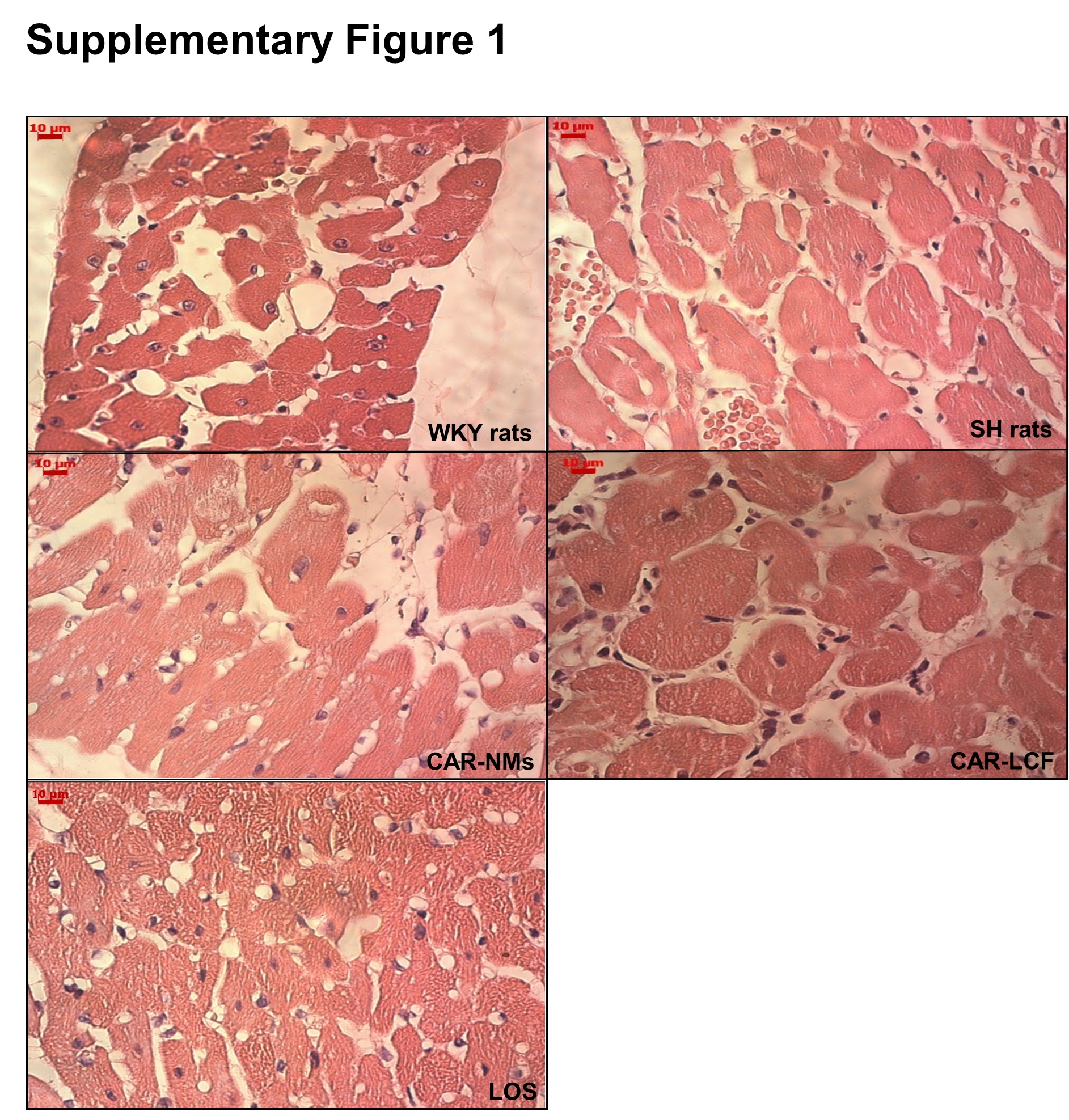

Supplement: Supplementary file 1 — Figure S1. Representative images of LV slices stained with hematoxylin–eosin. Hematoxylin–eosin staining was used for the analysis of cardiomyocyte surface area in LV of Wistar Kyoto rats (WKY rats) and spontaneously hypertensive rats after 8‐week treatment with vehicle (SH rats), 15 mg/kg carvedilol‐LCF (CAR‐LCF), 10 mg/kg losartan solution (LOS), or 15 mg/kg carvedilol Soluplus‐NMs (CAR‐NMs). (Original magnification 400×). LV, left ventricle. [file PRP2-13-e70125-s001.jpg]

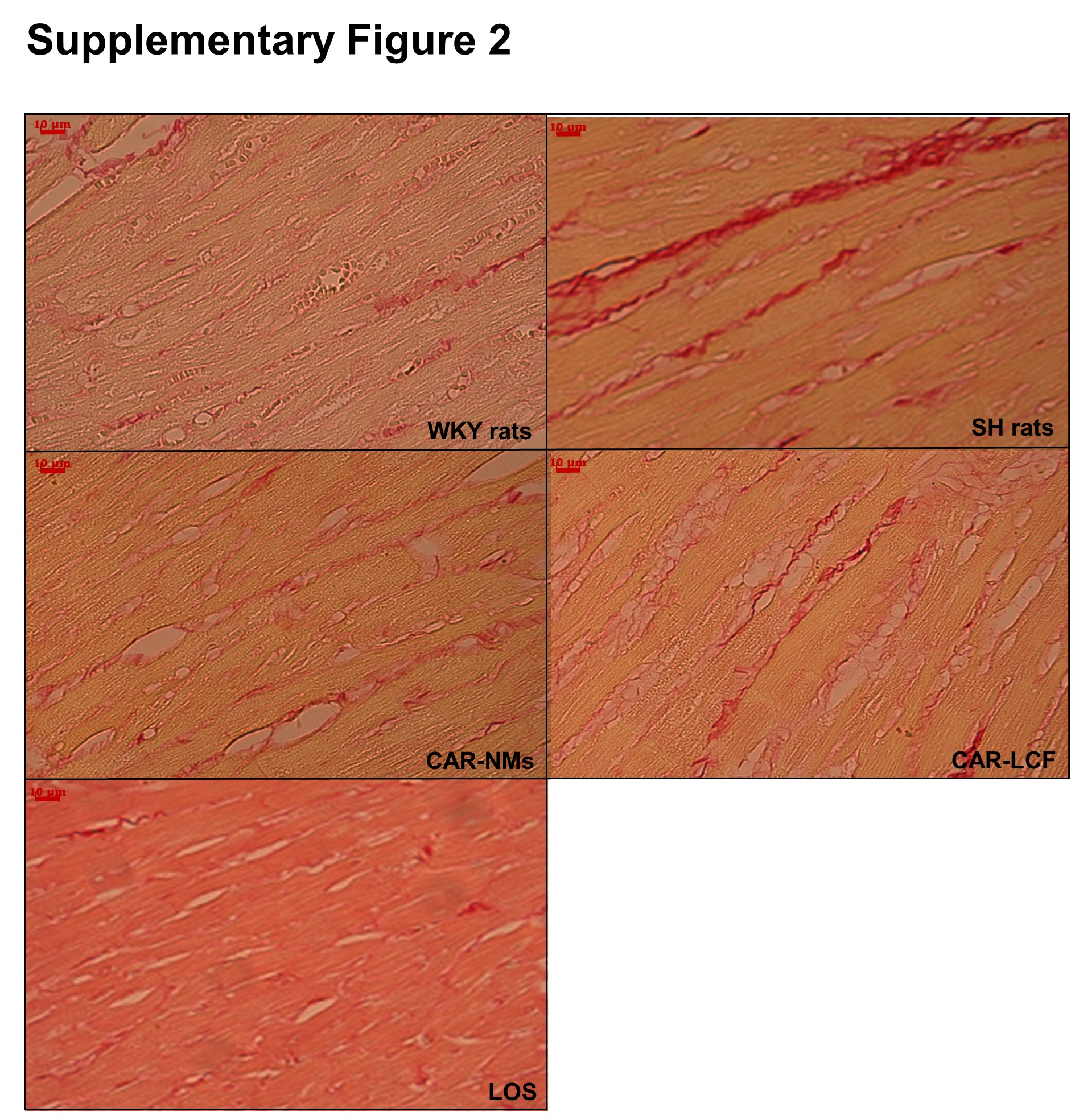

Supplement: Supplementary file 2 — Figure S2. Representative images of LV slices stained with Picrosirius Red. Picrosirius Red staining was used for the estimation of ICF in LV of Wistar Kyoto rats (WKY rats) and spontaneously hypertensive rats after 8‐week treatment with vehicle (SH rats), 15 mg/kg carvedilol‐LCF (CAR‐LCF), 10 mg/kg losartan solution (LOS), or 15 mg/kg carvedilol Soluplus‐NMs (CAR‐NMs). (Original magnification 400×). ICF, interstitial collagen fraction; LV, left ventricle. [file PRP2-13-e70125-s004.jpg]

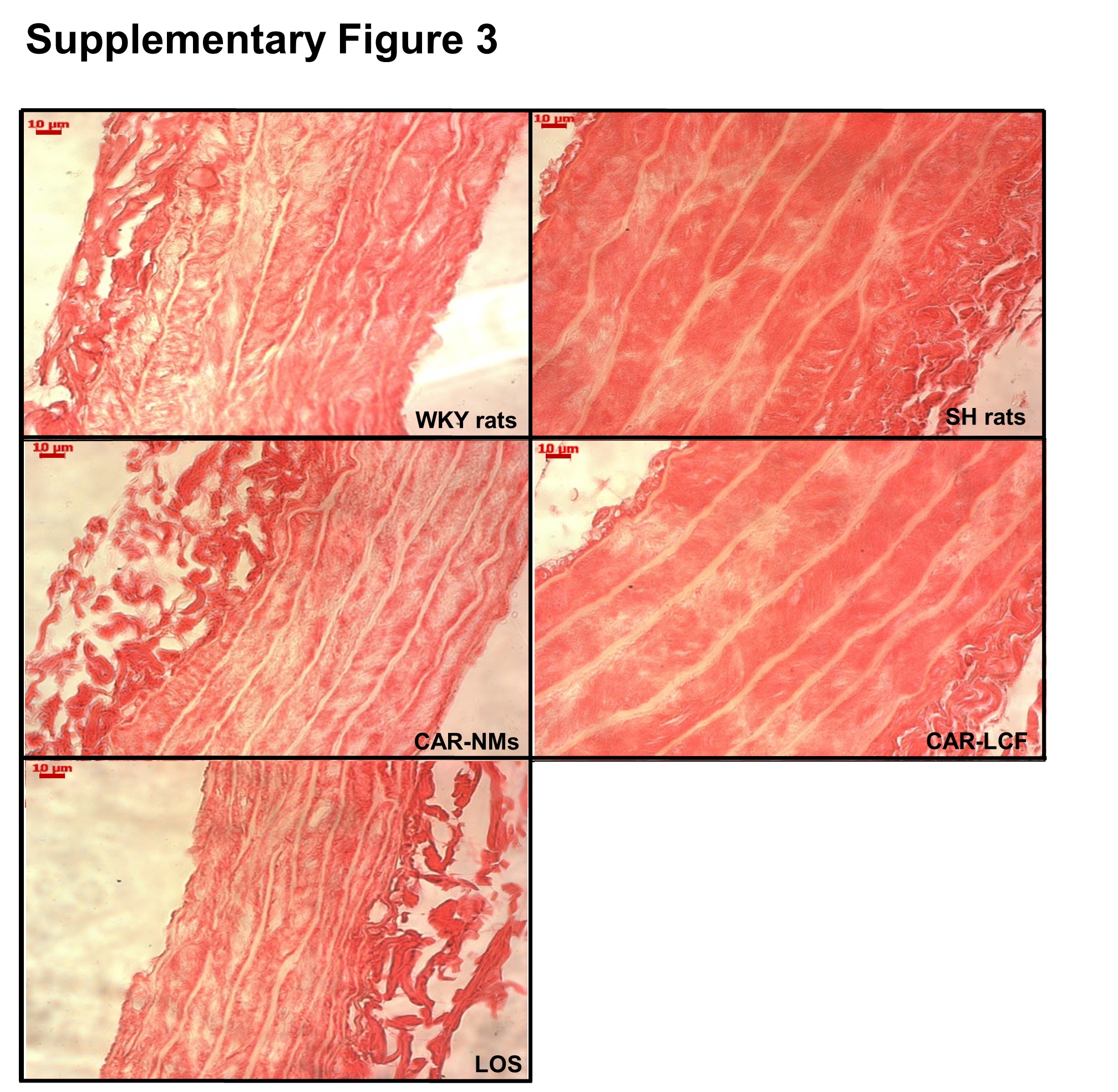

Supplement: Supplementary file 3 — Figure S3. Representative images of thoracic aorta rings stained with Picrosirius Red. Picrosirius Red staining was used for the estimation of ICF in thoracic aorta from Wistar Kyoto rats (WKY rats) and spontaneously hypertensive rats after 8‐week treatment with vehicle (SH rats), 15 mg/kg carvedilol‐LCF (CAR‐LCF), 10 mg/kg losartan solution (LOS), or 15 mg/kg carvedilol Soluplus‐NMs (CAR‐NMs). (Original magnification 400×). ICF, interstitial collagen fraction. [file PRP2-13-e70125-s003.jpg]

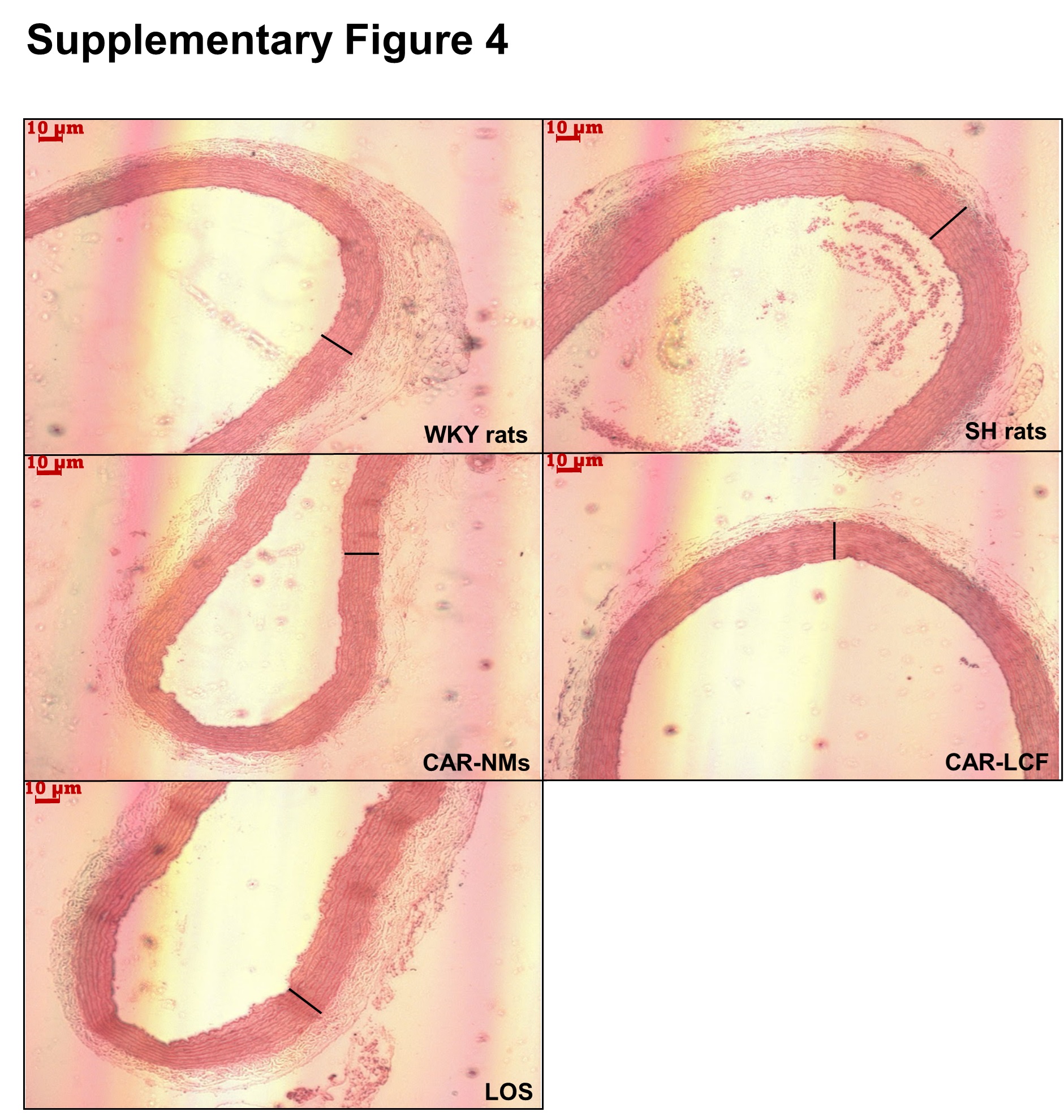

Supplement: Supplementary file 4 — Figure S4. Representative images of thoracic aorta rings stained with hematoxylin–eosin. Hematoxylin–eosin staining was used for the estimation of aortic wall thickness in Wistar Kyoto rats (WKY rats) and spontaneously hypertensive rats after 8‐week treatment with vehicle (SH rats), 15 mg/kg carvedilol‐LCF (CAR‐LCF), 10 mg/kg losartan solution (LOS), or 15 mg/kg carvedilol Soluplus‐NMs (CAR‐NMs). (Original magnification 40×). [file PRP2-13-e70125-s002.jpg]
